# Empowering senior medical residents as resuscitation team leaders

**DOI:** 10.1186/s12909-025-07240-5

**Published:** 2025-05-06

**Authors:** Florence Morriello, Jade Quirion, Homun Yee, Rashmi Narendrula

**Affiliations:** Northern Ontario School of Medicine University, Greater Sudbury, Canada

**Keywords:** Resuscitation, Leaders, Medical education

## Abstract

**Background:**

A code blue is a medical emergency indicating a patient requiring immediate attention with a systematic hospital wide response handled in a team based approach. In academic hospital settings, medical trainees are first responders to code blues. As first responders, a senior resident is required to assume the code blue leader role. Aim: This study explores what non-technical characteristics define a code blue leader to be a good leader?

**Methods:**

The study took place at the Northern Ontario School of Medicine. A qualitative methodology was applied. Semi-structured interviews were conducted sequentially.

**Results:**

Ten senior residents were interviewed using semi-structured interviews. Three distinct themes emerged, namely: individual factors, factors influencing team work and organization factors. Results confirm that residents lack confidence in leading code blue teams. This feeling is influenced by personal, team and situational factors. Residents lack training in non-technical skills and as a result feel they don’t know how to lead a code blue team and feel they lack the necessary skills to work effectively in a code blue team, especially under pressure.

**Conclusions:**

These data suggest that the lack of devoted training to non-technical skills, influences resident confidence, comfort, preparedness and functioning of resuscitative teams. Northern Ontario School of Medicine University REB approved, file number 6,021,198.

**Supplementary Information:**

The online version contains supplementary material available at 10.1186/s12909-025-07240-5.

## Background

Code blues are medical emergencies which are handled in a team based approach and require an effective team leader. “Code Blue” is a term generally used to indicate a patient requiring resuscitation or otherwise in need of immediate medical attention, most often as the result of a respiratory or cardiac arrest [1]. Every hospital, as part of a disaster plan, sets a policy to determine which departments provide personnel for code coverage. In theory, any medical professional may respond to a code, but in practice the team composition is limited to those who have advanced cardiac life support or other equivalent resuscitation training. Frequently, physicians trained in anesthesia, emergency medicine and internal medicine are charged with the task of leading these code blue teams.

Medical trainees are often the first responders to code blues. Medical trainees view code blue as challenging. In fact, leading a code blue resuscitation team is one of the greatest challenges a resident physician can face.[1, 2] Some of these challenges relate to the ability to work with a new team in acute circumstances. Often times poor team dynamics have an impact on personal performance and patient safety. Team dynamics are mostly influenced by team leaders. Research shows that an effective physician leader has the ability to improve teamwork[3], [4, 5, 6] and patient safety.[7, 8] Furthermore Rosenman underlines the importance of interpersonal skills and the ability to transition effectively as a team leader.[9] Team leaders are key players in a team, especially under acute circumstances. Team leaders help motivate others but also provide a sense of security for other team members even in situations where there is very little certainty while simultaneously helping to integrate interprofessional medical teams. Indeed the ability of leaders to evaluate and plan helps direct others toward a goal and every leader should have the ability to evaluate situations and make decisions accordingly.[10].

Code blue teams require a coordinated plan and someone who can effectively work with the interprofessional team in order to manage acute emergencies. The interprofessional team is typically composed of physicians, nursing staff, and a respiratory therapist. A senior resident, as a physician leader is expected to manage, lead and transfer competencies to the code blue team. Learners need to remain up-to-date on their knowledge, skills, and use critical thinking skills in order to delivery high-quality patient care.[11] Leaders further help nurture teamwork by integrating the work of physicians, nurses, and other health care professionals, with the ultimate goal of improving health care outcomes.[12] According to Rosenman et al., team leaders provide overall structure and direction to the team, monitor the task and other team members’ activities, coach team members as appropriate, and occasionally perform necessary hands-on patient care.[9] Interpersonal skills are essential in helping with conflict management but a team leader must also be a good communicator and collaborator. Research shows that team leaders are critical to a team and directly impact team performance and patient care. [13, 14] As such team leaders should be able to foster successful teamwork and allow team members to unlock their full potential and unique capacities.[15] While code leaders foster a culture of shared trust and respect, they must also create an environment where other interprofessional and team members feel encouraged to openly and effectively share ideas and contribute their expertise.

Team leadership behaviours are influenced by a number of factors including patient medical condition and stability, characteristics of team members (i.e. experience level, familiarity ), and environmental status (acute settings, uncontrolled conditions vs. controlled, stable conditions).[9] Team leadership becomes even more critical under uncertain and time-pressured conditions.[16] Leaders should be approachable, collaborate and provide direction to the remainder of team members even under critical or time-pressured conditions. Rosenman et al. (2016) point out that leadership is functional.[9] This suggests that a team leader assigns team members tasks and directs other team members when tasks are inadequately handled or not accomplished. As such, team leaders constant monitor other team members and transition from task to task effectively and efficiently while simultaneously coaching and integrating all team members in a task.

Code blue teams involve people of different training and skill sets. Leaders should possess behaviours that promote team cohesiveness, inspire and enable others to act effectively while putting forward their best abilities while possessing skills to help identify conflicts and help mitigate team harmony, especially under acute, time-pressured, situations. [9], [17, 18, 19] Through their ability to inspire and empower other team members, leaders are able to actualize group potential and help foster action-driven behaviour but also enable others to take responsibilities.[9], [17], [19].

Leadership skills become even more important when collaborating with interprofessional healthcare teams which is a competency expected of medical learners across the education continuum;[20], [21], [22] but attaining such proficiency is remarkably difficult. Despite the importance of leadership skills especially in uncertain, time-pressured conditions, dedicated leadership education is rarely part of a medical training program. There is an assumption that these competences can be acquired naturally without dedicated time to teach them and train learners.

In the past, medical educators and clinicians believed that medical trainees should only be taught technical skills in order to enable them to become effective code blue team leaders. Over the years, through direct observation and code blue debriefs, it became evident that trainees face challenges in assuming the role of a code blue leader despite successfully obtaining technical skills (knowledge of ACLS algorithm, skills to intubate, perform CPR). It has become evident that non-technical skills are equally important in becoming good leaders. Non-technical skills include, namely: (i) the ability to lead a team that can function together, (ii) a collaborator, (iii) a listener, (iv) a good communicator and (v) team player. [23], [24], [25] In order to understand the experiences and challenges in leading a code blue, former graduates of the internal medicine residency program at Northern Ontario School of Medicine University (NOSM U) were interviewed. The aim was to identify gaps in non-technical skills and ways to improve the education and training of residents in becoming effective code blue team leaders.

Specifically (a) what are the non-technical characteristics of a good code blue leader? (b) what are the experiences of code blue leaders? (c) what factors are perceived by senior internal medicine residents to influence the functioning of code blue teams?

## Methods

Perspectives and experiences of internal medicine residents at NOSM U, a Canadian University, were assessed in order to explore leadership skills and behaviours using a qualitative methodology. This study was grounded in a constructivist research paradigm, which acknowledges that knowledge is co-constructed through experiences and social interactions. Individual semi-structured interviews facilitated deeper discussion of more complex questions allowing participants to share detailed accounts of perceptions of their experiences, interpretations and perspectives of their code blue experiences. By embracing a constructivist approach, the study aimed to capture the nuanced and subjective realities of senior medical residents, recognizing that leadership development is shaped by personal and contextual factors.

### Instruments

Interviews with residents were carried out using a semi-structured format and were conducted only once. Interviews were either in-person or through zoom. Interviews lasted from 30 min to an hour. Interviews were recorded. The study data is stored on a hard drive. It is password protected and therefore has an audit trail and can be confirmed.

### Data availability

The datasets generated and/or analysed during the current study are not publicly available due to reasons of confidentiality and sensitivity. However, data are available from the corresponding author on reasonable request. All data are securely stored in controlled access data storage at the Northern Ontario School of Medicine University.

### Ethics approval and consent to participate

The study obtained Research Ethics Approval through NOSM U, Research Ethics Board file number 6021198. This research was conducted in full compliance with the Declaration of Helsinki. Written informed consent was obtained from all participants before participation in the study.

### Setting

The study took place at NOSM U, at the two main campuses: Health Sciences North (HSN), in Sudbury, and Thunder Bay Regional hospitals (TBRH), in Thunder Bay. HSN and TBRH are teaching hospitals affiliated with NOSM U and have a total of 454 and 395 beds respectively. Internal medicine residents at both sites are expected to have obtained Advanced Cardiac Life Support (ACLS) certification prior to residency and to renew certification as necessary. The ACLS is a resuscitation course which uses simulation-based learning strategies to teach and review intervention of cardiopulmonary arrest or other cardiac-related emergencies. Senior internal medicine residents (SIM) in their post-graduate year 2 and 3 are expected to lead code blues and manage code blue teams effectively. There is a monthly schedule and SIM are assigned as code blue leaders sporadically throughout the month.

Typically from 9:00 am to 5:00 pm, Monday through to Friday the code blue team includes: (1) at least 2–3 residents, a senior resident leading the code blue team and 1–2 junior residents to help with CPR and help with the code, (2) a respiratory therapist, (3) 3–4 nursing staff, (4) the most responsible physician, (5) an anesthesia resident or staff member and most times (6) a Critical care staff physician.

After 5:00 pm, the code blue team has 2–3 nursing staff and typically only one resident which is the SIM and a respiratory therapist. On holidays and weekends, the code blue team has 2 nurses, a SIM, a respiratory therapist and sometimes an emergency staff physician or critical care physician.

### Design and data collection

Qualitative exploratory methodology was chosen to explore participants’ (SIM) perspectives and expectations derived from their experiences. Using purposive sampling current SIM and recent graduates from 2022 until 2024 from NOSM U were contacted via email which outlined study details. Written informed consent was obtained from all participants prior to inclusion. A total number of 18 residents were contacted and 10 SIM residents volunteered agreed and were recruited in the study.

Participants were invited to undergo semi-structured standardized interviews using an interview guide of open-ended questions (appendix). In person or virtual interviews were held on a one-to-one basis and took 45 minutes to one hour to complete. All interviews were tape recorded and transcribed verbatim in order to limit potential note taking errors. [26] Interview questions (appendix) were aimed at understanding team leadership and team work. In particular, assessing the dynamic nature of the team leaders and their non-technical skills.

### Study duration

The qualitative study took five to six months to complete.

### Analysis

Transcripts were analyzed using framework analysis. The data was examined for patterns and shared meaning of data which were then categorized into ‘thematic’ codes. Through this process the researchers identified key themes and concepts which were used to develop ‘thematic’ codes. Transcribed interviews were descriptively coded by two independent reviewers. A coding template was developed after coding an initial set of interviews and this template was later used to code the remaining transcripts. A thematic analysis of the codes was conducted in order to create a summary report. Once all the codes were generated they were compared and reviewed. Disagreements between the two independent coders were reviewed by a third researcher, who facilitated discussions to reach a consensus.

### Results

In total 10 interviews were conducted. 60% of the participants were males and 40% were females. Seven participants had either completed the NOSM U internal medicine residency program and three participants were in their post graduate year 3 of training. Nine participants were Canadian medical graduates and one was an international medical graduate.

Three distinct themes emerged from the interviews. These related to individual factors, situational factors and factors influencing team work.

### Individual factors

Participants expressed a number of negative and positive emotions relating to their experience with code blues. SIM felt anxious, uncertain, embarrassed and fearful when arriving to a code blue and throughout code blues. Other SIM felt awkward, scrutinized, unequipped, unwelcomed and angry during code blues for not always knowing what to do during the resuscitation.

*“To me*,* Code Blues are scary and awkward*,* I feel that I don’t know what to do and lack the clinical knowledge and practice.” (Internal Medicine Postgraduate resident Year 3)”*.

SIM, unanimously felt that they had received very little mentorship or guidance by staff members. They felt it would have been beneficial to work more closely with staff members as leaders of code blue. They thought they would have gained more confidence if staff physician, if present, would have allowed them to lead the code blues with them providing support and guidance.

SIM felt these feelings of insecurity were compounded when they were faced with complex patients, patients with unclear resuscitation status, patients with prolonged hospitalizations and patients who lacked documentation of their hospital course. If SIM had had prior clinical encounters with patients, they felt more at ease when leading the code blue on that patient.

Prior experience leading code blues directly impacted residents when faced with the challenging of leading a code blue. In fact, the lack of code blue experience negatively affected resident’s confidence when arriving to a code blue setting. Participants felt that the challenge of leading code blues and trying to manage a code blue team was inversely proportional to SIM’s experience and training.

When you haven’t led a code blue and suddenly after six months from the time you completed your ACLS you are delegated to lead one, it is a scary situation.

Residents also underlined the cognitive burden when attending code blues. They believe this may be due to infrequent exposure to code blues, impacting on their experience and uncertainty when leading code blues. On a whole, residents felt that ACLS training was somewhat helpful but not sufficient.

*“ACLS is helpful but I did practice leading a code until almost 5 months later and felt lost (Internal Medicine Postgraduate resident Year 3)”*

*“Working with new code blue teams is sometimes challenging and crowd control is not easy.”*

Situational factors outlined in the interviews included: (i) location of the code blue, (ii) available resources and environment, (iii) Timing of the code blue.

#### Location

Participants felt that location of the code makes a difference with respect to the overall team function of a code blue. ‘Some areas of the hospital are difficult to get to’ and this impacted the arrival time of the code team. Room space plays a role on the code blue. For example, patients in multi-patient rooms with limited space makes it challenging for the code blue team to bring in the crash cart and the code blue leader to lead the code. Similarly, patients who are bed-spaced into a hallway creates challenges for the code blue team. For these patients, code leaders need to be mindful of privacy issues but also ensure that the team stays focused and crowds are controlled.

#### Available resources and environment

The type of resources available impacts the function of a code blue team, in particular affecting the leader. In fact, SIM felt that ‘trying to lead a code on a patient who is physically in the main entrance of the hospital where resources are limited is a nightmare’. It is easier to assign tasks to the team and lead code blues when patients are in private rooms. Similarly if ‘we need to draw blood, blood tube collection vials are easily available on a unit, unlike in a hallway or in a crowded main entrance.’

In fact, patients in room are better monitored during code blues. ‘A patient in a room has a monitor which can be temporarily used while setting up the code monitor and defibrillator whereas for bed spaced patients this is impossible.’ Also when a patient is lying in bed, it is easier to place the code board beneath him/her and immediately start chest compressions whereas patients who code in a cafeteria, hallway this is more challenging. All this impacts team dynamics and the ability to lead the code blue as a good leader.

Although rare, when multiple code blues are simultaneously occurring, resources for the code blue team become extremely limited. The crash cart is kept in a different location than its usual place on the unit. A second code blue team is activated and this team is smaller with fewer members (a second SIM and usually 1 nurse, a second respiratory therapist).

“I had to run a second code blue with virtually no help-I had one nurse and a respiratory therapist which I was using to help with chest compressions. Luckily an anesthesia resident also came to help with airway management.”

SIM felt that large open uncontrolled spaces such as hallways, radiology department or crowded areas are the most challenging and scary. They felt that you ‘can’t control what is going on.’ There are a lot of people and everyone is speaking over each other. In fact they felt it’s difficult for a code leader to keep the team together.

*“I remember some code blues on surgical wards*,* some of the staff weren’t as familiar with code blue. It could get chaotic with 10–20 people in the room*,* everyone is looking around not sure what to do. Hard to get the group focused and running an efficient code blue.”*

#### Timing of the code blue

The day of the week and time of day impacts a code blue team. In fact, time of day and day of week makes a difference in code blue team composition and the functionality of a code blue. Typically from 9:00 am to 5:00 pm, Monday through to Friday there are plenty of people around to help with a code blue. Whereas with less members available to support the code team, the function and dynamics of the code blue are different. During the day hours, residents felt they received more guidance, teaching and support when at code blues. On the contrary, SIM felt that ‘fewer people were around to help with code blues which makes it challenging to lead a code, especially in your first month transitioning from a junior resident to a senior one with no prior experience leading a code. You need to depend on your code blue team and if that is a skeleton, it is challenging to lead a code blue team’.

#### Team dynamics and communication

Code blue teams vary in skills sets and expertise. One of the most important factors is team composition and ensuring that there is at least one person who can lead a code blue. Prior history of working experiences with people on the code blue team impacts the function of the code blue team. In fact, SIM felt “if one has already worked with a code blue nurse or respiratory therapist in the past, they will know their names and this will help with communication and the flow of the code blue.” Comfort level among team members working within a particular code blue team makes it easier to work together and this further impacts the code blue leader’s effectiveness.

Communication plays an important role on team function. On arrival to a code blue, a team leader should let everyone know that he/she is leading the code. He or she must establish roles on the code blue team. Establishing a code blue leader is important for team dynamics. At times when a code blue leader does not identify him/herself, team members may become confused and not know who is leading the code.

The type of communication used in code blues influences the team members and the dynamics of the team. For example closed loop communication in addition to addressing members of the team by name helps delegate roles and helps to keep the code blue and the workflow organized. [27] On the other hand, bad communication or lack of clear communication creates confusion and increases anxiety levels among code blue team members. A code blue leader who does not adopt closed loop communication makes the code blue team very dysfunctional and may impact patient outcome.[27]

*“When people are new and don’t have code experience they often don’t use closed loop communication so it’s harder to determine who has what role when closed loop communication is not being used you don’t know who’s carrying out your orders or if they are being carried out at all.”*

As a code blue team leader it is important to arrange and lead debriefing sessions post code blues. In fact, debriefing is an essential part of a code blue.[28]–[29] Residents felt that timely discussion of a code blue adds to team bonding but also fosters personal learning and growth and improves teamwork. Residents described their experiences as negative as most of the code blues they attended, there was seldomly a debrief post code blues.

“Only one debrief, and that was after a really bad outcome. There were a lot of emotions running high, the nursing staff and other team members wanted to debrief and the staff was in attendance as well. I think it was really helpful for everyone to deconstruct the whole situation, but that was the only time that happened.”

‘Lack of debrief negatively impacts learning and growth for the code blue teams.’

## Discussion

Our study shows that effective management of patients in cardiac arrest requires a combination of personal knowledge and skills, team competencies and understanding situational factors. Required competencies of a code blue leader include the individual factors, timing and location of code blues, available resources and team dynamic skills to support the collective efforts of team members in order to deliver optimal patient care during code blues.

The aim of this study was to explore the resident experiences with leading code blues. Our study results suggest that residents lack the skills and confidence to lead code blues. This is attributed to predominately lack of non-technical skills including good communication, leading and working with a code blue team and understanding the environment and organizational factors. The study highlights the importance of non-technical skills on code blue teams and leaders. Results suggest leaders with good non-technical skills have more confidence when in a team environment, are better able to guide a team under pressure and overall are better leaders. This is supported by a previous study which found that non-technical skills redefine surgical success under pressure and enable leaders to lead with confidence, and manage stress.[30] These are all skills that extend beyond just knowledge. Similar to surgery, code blues are high stress environments that require the knowledge of technical skills but equally important are non-technical especially for a code blue leader.

Our results indicate that residents often feel unprepared to lead code blues due to gaps in their non-technical skills such as communication, leadership, and environmental awareness. This lack of preparedness leads to decreased confidence in their ability to effectively manage the code blue team under pressure, which ultimately affect patient care.

One potential solution to address the gaps identified in this study is the implementation of simulation-based training programs are increasingly used to help residents practice leading code blues in a controlled, low-risk environment. These simulations expose residents to high-pressure scenarios without the risk of patient harm. However, our study suggests that despite these efforts, residents continue to feel unprepared, indicating that simulation alone may not be sufficient. To address this, simulation scenarios should not only focus on technical skills but also provide opportunities for residents to practice communication, leadership, and stress management in a realistic, high-pressure context.

Additionally, implementing mandatory debriefings after code blue events may also be beneficial to our residents. These debriefings provide an opportunity for residents to reflect on their performance, discuss challenges, and receive feedback from staff physicians. Some hospitals have already incorporated debriefing sessions, which have proven effective in enhancing both technical and non-technical skills, improving communication, reducing stress, and boosting team confidence.[9], [31].

### Consideration of leadership roles in code blues at academic institutions

The findings of this study raise the question of whether it is always appropriate for residents to lead code blues, especially if they feel unprepared. In some healthcare settings, experienced physicians, such as attending physicians or fellows, take the lead during code blue events, thereby reducing stress and pressure on residents. Given the results of our study, it may be worth reconsidering whether residents should be assigned leadership roles in these high-stress situations or if more experienced team members should take charge to ensure optimal patient care. Further research is needed to explore the impact of different leadership models on team dynamics and patient outcomes during code blue events.

### Practical implications

This study has several practical implications for improving personal performance, team collaboration and situational awareness during code blues, ultimately reducing cognitive load for residents. The results support the incorporation of formal, non-technical skills training into the postgraduate medical curriculum. This could help address challenges such as limited exposure to code blue events, foster better team collaboration, and enhance residents’ troubleshooting abilities. By integrating non-technical skills into the curriculum, residents can be better prepared to lead in high-pressure situations. These study results can be applied to various educational settings and institutions.

### Limitations

While this study provides valuable insights into the challenges faced by residents in leading code blues, it has several limitations. The sample size may not fully represent the diversity of experiences across different residency programs, and the self-reported nature of the data could introduce response bias, as residents who have faced challenges might be more likely to participate. Future studies should aim for a larger, more diverse sample and include objective performance measures, such as direct observation during code blue simulations. Furthermore, the cross-sectional nature of this study limits our ability to establish causal relationships between residents’ perceived lack of non-technical skills and their actual performance during code blue events.

## Conclusions

This study highlights the significant impact of non-technical skills on improving personal performance, team collaboration, and situational awareness during code blue events. By reducing cognitive load for residents, structured training in these skills can enhance decision-making and leadership in high-pressure situations. The findings support integrating formal non-technical skills training into postgraduate medical education, addressing challenges such as limited exposure to real-life emergencies and the need for effective team coordination. Implementing such training across various educational settings and institutions can improve patient outcomes and resident preparedness for emergency scenarios.

## Electronic supplementary material

Below is the link to the electronic supplementary material.

Supplementary Material 1

Supplementary Material 2

## Data Availability

The datasets generated and/or analysed during the current study are not publicly available due to reasons of confidentiality and sensitivity. However, data are available from the corresponding author on reasonable request. All data are securely stored in controlled access data storage at the Northern Ontario School of Medicine University.

## Abbreviations

REB: Research ethics board
NOSM: U Northern ontario school of medicine university
IM: Internal medicine
ACLS: Advanced cardiovascular life support
SIM: Senior internal medicine

## References

[1] Eroglu SE, Onur O, Urgan O, Denizbasi A, Akoglu H. Blue code: is it a real emergency? World J Emerg Med. 2014;5(1):20. 25215142 10.5847/wjem.j.issn.1920-8642.2014.01.003 PMC4129865

[2] Fee-Mulhearn AL, DePriest J, Teleron A. A novel ACLS team leader checklist implemented to improve resuscitation efforts. Resuscitation, 2013;84(9):e115. 10.1016/j.resuscitation.2013.03.002 23558006

[3] Curry SE, Cortland CI, Graham MJ. Role-modelling in the operating room: medical student observations of exemplary behaviour. Med Educ. 2011;45(9):946–57. 21848723 10.1111/j.1365-2923.2011.04014.x

[4] Nembhard IM, Edmondson AC. Making it safe: the effects of leader inclusiveness and professional status on psychological safety and improvement efforts in health care teams. J Organizational Behavior: Int J Industrial Occup Organizational Psychol Behav. 2006;27(7):941–66.

[5] Rehim SA, DeMoor S, Olmsted R, Dent DL, Parker-Raley J. Tools for assessment of communication skills of hospital action teams: a systematic review. J Surg Educ. 2017;74(2):341–51. 27771338 10.1016/j.jsurg.2016.09.008

[6] Brewster DJ, Butt WW, Gordon LJ, Rees CE. Leadership in intensive care: A review. Anaesth Intensive Care. 2020;48(4):266–76. 32741196 10.1177/0310057X20937319

[7] Baker DP, Gustafson S, Beaubien J, Salas E, Barach P. Medical teamwork and patient safety: the evidence-based relation. AHRQ Publication. 2005;5(53):1–64.

[8] Sutcliffe KM, Lewton E, Rosenthal MM. Communication failures: an insidious contributor to medical mishaps. Acad Med. 2004;79(2):186–94. 14744724 10.1097/00001888-200402000-00019

[9] Rosenman ED, Branzetti JB, Fernandez R. Assessing team leadership in emergency medicine: the milestones and beyond. J Graduate Med Educ. 2016;8(3):332–40. 10.4300/JGME-D-15-00400.1 PMC4936849 27413434

[10] Kilminster S, Cottrell D, Grant J, Jolly B. AMEE guide 27: effective educational and clinical supervision. Med Teach. 2007;29(1):2–19. 17538823 10.1080/01421590701210907

[11] McCallin A. Interdisciplinary team leadership: a revisionist approach for an old problem? J Nurs Adm Manag. 2003;11(6):364–70. 10.1046/j.1365-2834.2003.00425.x 14641717

[12] Hunziker S, Johansson AC, Tschan F, Semmer NK, Rock L, Howell MD, Marsch S. Teamwork and leadership in cardiopulmonary resuscitation. J Am Coll Cardiol. 2011;57(24):2381–8. 21658557 10.1016/j.jacc.2011.03.017

[13] Burke CS, Sims DE, Lazzara EH, Salas E. Trust in leadership: A multi-level review and integration. Leadersh Q. 2007;18(6):606–32.

[14] Kozlowski SW, Ilgen DR. Enhancing the effectiveness of work groups and teams. Psychol Sci Public Interest. 2006;7(3):77–124. 26158912 10.1111/j.1529-1006.2006.00030.x

[15] Lieff SJ, Yammarino FJ. How to lead the way through complexity, constraint, and uncertainty in academic health science centers. Acad Med. 2017;92(5):614–21. 28441672 10.1097/ACM.0000000000001475

[16] Janssens S, Simon R, Beckmann M, Marshall S. Shared leadership in healthcare action teams: A systematic review. J Patient Saf. 2021;17(8):e1441–51. 29870514 10.1097/PTS.0000000000000503

[17] Outhwaite S. The importance of leadership in the development of an integrated team. J Nurs Adm Manag. 2003;11(6):371–6. 10.1046/j.1365-2834.2003.00427.x 14641718

[18] Anonson JM, Ferguson L, Macdonald MB, Murray BL, Fowler-Kerry S, Bally JM. The anatomy of interprofessional leadership: an investigation of leadership behaviors in team‐based health care. J Leadersh Stud. 2009;3(3):17–25.

[19] Thompson G, Glasø L. Situational leadership theory: A test from three perspectives. Leadersh Organ Dev J. 2015;36(5):527–44.

[20] WFME. Continuing professional development of medical Doctors. Copenhagen (Denmark). World Federation for Medical Education 2015.

[21] ACGME. 2019. Chicago, Illinois: Accreditation Council for Graduate Medical Education. [accessed 2024 Aug 12]. https://www.acgme.org

[22] LCME. Liaison Committee on Medical Education. [accessed 2024 Sep 11]. 2019.

[23] Black A, Brown O, Utunen H, Gamhewage G, Gore J. Insights on public health professionals non-technical skills in an emergency response (multi-team system) environment. Front Psychol. 2022;13:827367. 35774938 10.3389/fpsyg.2022.827367 PMC9239737

[24] Peddle M, Bearman M, Radomski N, Mckenna L, Nestel D. What non-technical skills competencies are addressed by Australian standards documents for health professionals who work in secondary and tertiary clinical settings? A qualitative comparative analysis. BMJ Open, 2018;8(8):e020799. 10.1136/bmjopen-2017-020799 PMC6078249 30082346

[25] Porter JE, Cant RP, Cooper SJ. Rating teams’ non-technical skills in the emergency department: A qualitative study of nurses’ experience. Int Emerg Nurs. 2018;38:15–20. 29422222 10.1016/j.ienj.2017.12.006

[26] DeJonckheere M, Vaughn LM. Semistructured interviewing in primary care research: a balance of relationship and rigour. Family Med Community Health, 2019;7(2). 10.1136/fmch-2018-000057 PMC6910737 32148704

[27] Spitzer CR, Evans K, Buehler J, Ali NA, Besecker BY. Code blue pit crew model: A novel approach to in-hospital cardiac arrest resuscitation. Resuscitation. 2019;143:158–64. 31299222 10.1016/j.resuscitation.2019.06.290

[28] Price JW, Applegarth O, Vu M, Price JR. Code blue emergencies: a team task analysis and educational initiative. Can Med Educ J, 2012;3(1):e4. PMC4563641 26451171

[29] Schober P, Kistemaker KR, Sijani F, Schwarte LA, van Groeningen D, Krage R. Effects of post-scenario debriefing versus stop-and-go debriefing in medical simulation training on skill acquisition and learning experience: a randomized controlled trial. BMC Med Educ. 2019;19:1–7. 31488113 10.1186/s12909-019-1772-y PMC6727540

[30] Cavallo SM, Pellencin E, Carone G, Castelli N, Ayadi R, Olldashi F, Perin A. Non-technical skills for neurosurgeons: an international survey. Brain Spine, 2024;102923. 10.1016/j.bas.2024.102923 PMC11408938 39296491

[31] Clarke S, Apesoa-Varano EC, Barton J. Code Blue: methodology for a qualitative study of teamwork during simulated cardiac arrest. BMJ Open, 2016;6(1):e009259. 10.1136/bmjopen-2015-009259 PMC4716199 26758258

