# Supplementary material for: Empowering senior medical residents as resuscitation team leaders
Source: BMC Med Educ. 2025 May 6;25:662. doi: 10.1186/s12909-025-07240-5 (PMC12057051; doi:10.1186/s12909-025-07240-5)
Supplement: Supplementary file 1 — Supplementary Material 1 [file 12909_2025_7240_MOESM1_ESM.docx]

**Appendix: Interview Questions:**

1. How many times over the last 24 months were you a Code Blue Team Leader?
2. From what you can recall were most of the Code Blues that you were the leader during the week or on the weekend?
3. From what you can recall were most of the Code Blues that you were the leader at night (from 17:01-7:59) or during the day (from 8:00-17:00)?
4. Where did the Code Blues occur? On the ward, in the step-up unit?
5. Can you describe a Code Blue that went really well? Were there any particular aspects you feel may have contributed to this?
6. Can you describe a Code Blue that you feel did not go as well as you had anticipated? Why do you think this may have happened? Lack of support? Location? Time of day?
7. Based on your experience do you find that the Code Blue teams run well? Are you always able to identify the members of the team?
8. Based on your experience what characteristics should a Code Blue team leader possess?
9. As a code team leader, do you sometimes find it difficult to work with some of the team members? Nursing staff? Respiratory therapist? Why? Is it because of lack of communication, conflict?
10. How do you perceive your role as a Code Blue leader, are you well prepared? Is there any additional training you would find helpful?
