# Supplementary material for: Empowering senior medical residents as resuscitation team leaders
Source: BMC Med Educ. 2025 May 6;25:662. doi: 10.1186/s12909-025-07240-5 (PMC12057051; doi:10.1186/s12909-025-07240-5)
Supplement: Supplementary file 2 — Supplementary Material 2 [file 12909_2025_7240_MOESM2_ESM.docx]

Study Title: Code Blue Team Leaders, ‘Empowering Senior Residents as Code Blue Leaders’

**Participant Statement of Consent**

By signing this consent form, I acknowledge that:

- This research study has been explained to me, and my questions have been answered to my satisfaction.
- I have been informed of the alternatives to participation in this study.
- I know that I have the right not to participate and the right to withdraw from this study without affecting the medical care received at, employment at, or other relationship with the Northern Ontario School of Medicine University now or in the future for me or my family.
- The potential risks and benefits (if any) of participating in this study have been explained to me.
- I understand that the text published in the article will be available on the internet and may be seen by the general public.
- I know that I may ask, now or in the future, any questions I have about this study.
- I have been told that information about me and my participation in this study will be kept confidential and that no personally identifying information will be disclosed without my permission unless required by law.
- I have been given sufficient time to read the information in this consent form.
- I will be given a signed and dated copy of this consent form.

I give my consent to participate in this study and for information about myself to be published in a medical journal. Signing this consent form does not remove my rights to privacy.

|  |  |  |  |  |  |  |
| --- | --- | --- | --- | --- | --- | --- |
| Participant name (print) |  | Participant signature |  | Date |  | Time |

I have explained to the above-named participant the nature and purpose, the potential benefits, and possible risks of participation in this study. All questions that have been raised about this study have been answered.

|  |  |  |  |  |  |  |  |  |
| --- | --- | --- | --- | --- | --- | --- | --- | --- |
| Name of person obtaining consent (print) |  | Position/Title of person obtaining consent (print) |  | Signature of person obtaining consent |  | Date |  | Time |
